# Molecular interactions between single layered MoS_2_ and biological molecules†
†Electronic supplementary information (ESI) available: SFG data analysis methods, spectral fitting parameters, additional spectra, CD spectrum, and details about MD simulation methods. See DOI: 10.1039/c7sc04884j


**DOI:** 10.1039/c7sc04884j

**Published:** 2017-11-30

**Authors:** Minyu Xiao, Shuai Wei, Yaoxin Li, Joshua Jasensky, Junjie Chen, Charles L. Brooks, Zhan Chen

**Affiliations:** a Department of Chemistry , University of Michigan , Ann Arbor , Michigan 48109 , USA . Email: zhanc@umich.edu

## Abstract

Two-dimensional (2D) materials such as graphene, molybdenum disulfide (MoS_2_), tungsten diselenide (WSe_2_), and black phosphorous are being developed for sensing applications with excellent selectivity and high sensitivity. In such applications, 2D materials extensively interact with various analytes including biological molecules. Understanding the interfacial molecular interactions of 2D materials with various targets becomes increasingly important for the progression of better-performing 2D-material based sensors. In this research, molecular interactions between several *de novo* designed alpha-helical peptides and monolayer MoS_2_ have been studied. Molecular dynamics simulations were used to validate experimental data. The results suggest that, in contrast to peptide–graphene interactions, peptide aromatic residues do not interact strongly with the MoS_2_ surface. It is also found that charged amino acids are important for ensuring a standing-up pose for peptides interacting with MoS_2_. By performing site-specific mutations on the peptide, we could mediate the peptide–MoS_2_ interactions to control the peptide orientation on MoS_2_.

## Abstract 2

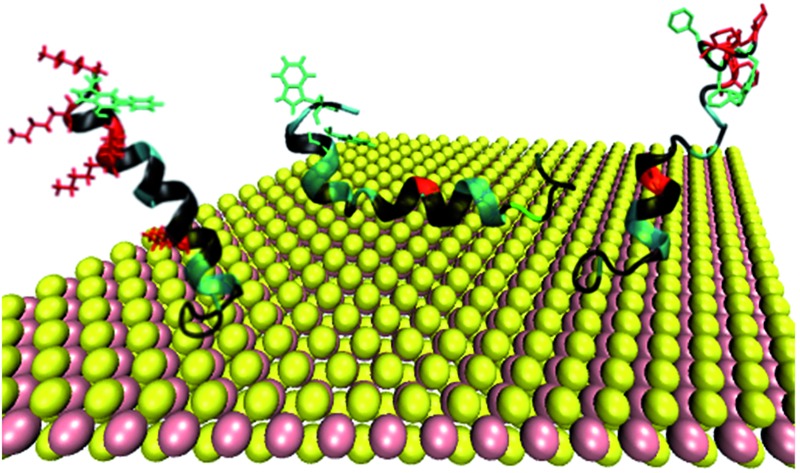
In this research, molecular interactions between several *de novo* designed alpha-helical peptides and monolayer MoS_2_ have been studied.

## Introduction

The unique properties of two-dimensional (2D) materials such as graphene allow ultra-sensitive electronic devices to be fabricated for gas sensing, biomolecular detection, *etc.*^1^,^2^ Molybdenum disulfide (MoS_2_), a representative 2D material, has been extensively used for sensing applications,^3^,^4^ including the construction of biosensors or for biomolecular detection.^5^–^7^ For example, previous research has reported on a MoS_2_-based fluorescence DNA sensor^5^ and the scalable production of MoS_2_ based biosensors with proteins.^7^ Biological molecules have also been reported to help facilitate the exfoliation of monolayer MoS_2_ flakes in the aqueous phase.^8^,^9^ We believe that it is therefore crucial to understand the interaction mechanisms between biological molecules and MoS_2_ surfaces in order to help the design of MoS_2_ based biological sensors.

Research has been performed to study molecular interactions between peptides/proteins and a MoS_2_ sheet using simulation methods,^10^–^12^ but experimental studies to validate such simulation results are rare. In this study, we chose an alpha-helical antimicrobial peptide, a hybrid of cecropin and melittin, as our model to investigate the molecular interactions with MoS_2_ using both molecular dynamics simulations and experiments.

**Fig. 1:**
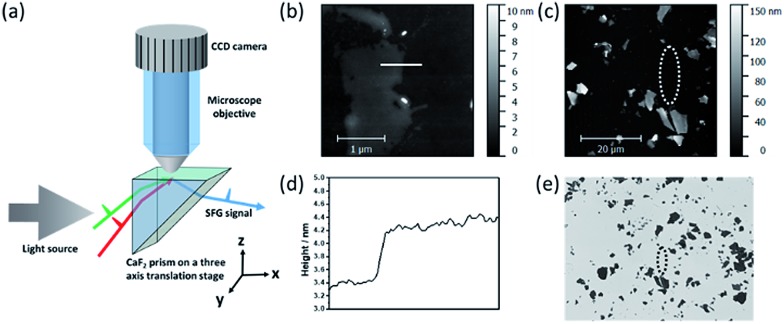
(a) Schematic of the optical microscope-SFG setup; (b and c) AFM images of mechanically exfoliated MoS_2_ flakes with different magnifications. (d) Thickness measured by AFM, indicating that the MoS_2_ flake in (b) is a monolayer. (e) Optical image of MoS_2_ on a CaF_2_ prism surface (the circle is the focus of the visible beam for SFG data collection). According to the positions of the multilayered MoS_2_ flakes below the circle in both AFM (c) and optical (e) images, we can identify the monolayer MoS_2_ sample in the circle.

To probe the interactions between the alpha-helical hybrid peptide and a monolayer MoS_2_ surface, we implemented optical microscopy with sum frequency generation (SFG) vibrational spectroscopy (Fig. 1). While optical microscopy can help us locate the positions of monolayer MoS_2_ flakes, SFG can enable the study of peptide/MoS_2_ interactions *in situ* with monolayer sensitivity. More details about the microscope-SFG setup can be found in the ESI.†

A MoS_2_ monolayer was not visible on a CaF_2_ substrate. To locate such a monolayer region, it was necessary to pre-determine a location of the monolayer MoS_2_ flake by AFM, using thick multilayered MoS_2_ regions nearby as fiducial markers (Fig. 1). Then the fiducial markers were located by SFG microscopy, and the visible and infrared beams were focused onto the nearby MoS_2_ monolayer region to collect the SFG spectra (Fig. 1).

SFG is a second order nonlinear optical spectroscopic technique with excellent surface selectivity; its equipment and experimental details have been published and will not be repeated here.^13^–^21^ SFG has been extensively applied to investigate molecular interactions at various interfaces, including interfaces involving polymers, water, DNA, peptides and proteins.^17^–^19^,^22^–^28^ Specifically, for alpha-helical peptides, an SFG amide I peak centered near 1650 cm^–1^ can be detected. The orientation of a single alpha helical peptide at an interface can be described by a tilt angle *θ* (the angle between the peptide main axis and the surface normal). Through analysis of the SFG amide I spectra collected using different combinations of p and s polarizations of the input/output beams, the tilt angle *θ* can be deduced by the measured *χ*_ppp_/*χ*_ssp_ ratio.^29^

## Materials and methods

### Peptide sequence and experimental conditions

All MoS_2_ samples were prepared on right-angled CaF_2_ prisms through a mechanical exfoliation method. All peptide mutants were purchased from Peptide 2.0 and were used as received, and all peptide solutions were prepared at a concentration of 1.0 μM. Sequences of all peptides used were: wild-type hybrid peptide (KWKLFKKIGIGAVLKVLTTGLPALIS), mutant A (KAKLAKKIGIGAVLKVLTTGLPALIS), mutant B (SWSLFSSIGIGAVLKVLTTGLPALIS), and mutant C (KWKFFKKIGIGAVLKVLTTGLPALIS).

### Brief description of microscope-SFG

Details about the microscope-SFG setup have been published previously.^14^ Briefly, to allow enough working space for an optical microscopic system, an “inverted” total internal reflection SFG sample geometry was used in this study (Fig. 1). Both the visible (532 nm) and tunable infrared (IR) beams were spatially and temporally overlapped onto the prism surface using two CaF_2_ lenses. A right-angled CaF_2_ prism substrate was placed on a three-axis translational stage: both positions on the *x*–*y* plane and the height of the sample can be fine-tuned. The optical microscopic system was positioned above the prism substrate to allow visual monitoring of the sample and the laser spot (for SFG signal detection) *in situ*. A 40× objective and telescope were used to magnify the image onto a CCD camera. While keeping the optical microscope stationary, the three-axis translational stage mentioned above allowed full freedom to move the sample to find an ideal position for data collection. Using AFM, we determined the location of a monolayer of MoS_2_ with the help of neighboring thick multilayered MoS_2_ regions (Fig. 1). Such a location of a monolayer MoS_2_ region could be identified using the microscope-SFG with the help of the multilayered MoS_2_ regions. Then both the visible and infrared beams were focused onto this region to collect SFG spectra (Fig. 1). Structural information such as the molecular orientation of surface peptides on a single layer of MoS_2_ could then be measured *via* SFG.

## Results and discussion

**Fig. 2:**
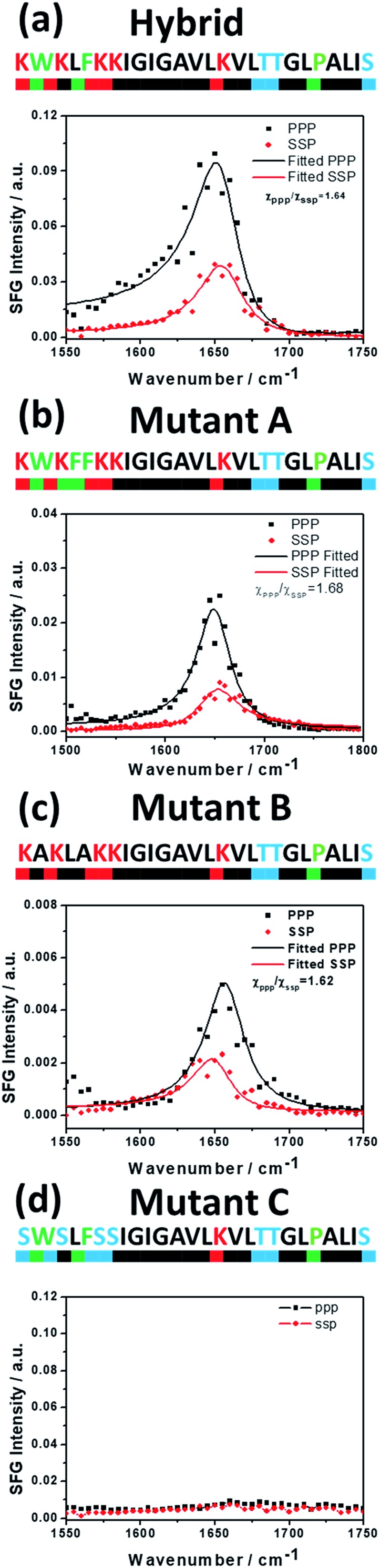
Primary sequence (top) and SFG spectra collected from the interface between MoS_2_ and solutions (bottom) of the native cecropin–melittin hybrid peptide (a), mutant A (b), mutant B (c) and mutant C (d).

The sequence of a native cecropin–melittin hybrid peptide is shown in Fig. 2, where charged, hydroxyl group-containing, hydrocarbon side chain-containing, or aromatic ring-containing amino acids are labeled in red (most hydrophilic), blue (hydrophilic), black (hydrophobic), and green (most hydrophobic) respectively.^30^

**Fig. 3:**
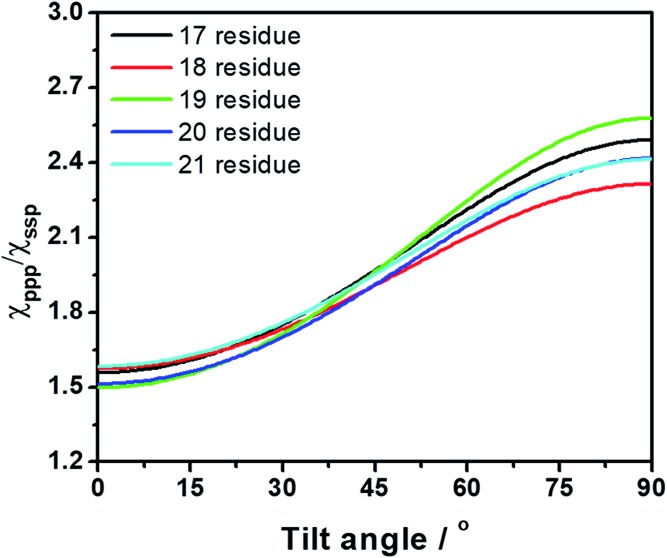
Dependence of the tilt angle on the measured SFG signal strength *χ*_ppp_/*χ*_ssp_ ratio for several different lengths of alpha-helical peptide (17–21 residues).

SFG ssp (s-polarized signal, s-polarized input visible, p-polarized input IR beams) and ppp spectra were collected from the single layer MoS_2_/hybrid peptide solution interface (Fig. 2a). Because no SFG signal could be detected from the bare CaF_2_/peptide solution interface (not shown), such signals must be due to the peptides on MoS_2_. Both SFG spectra exhibit a distinct amide I peak at 1650 cm^–1^, indicating that the hybrid peptide adopts an alpha-helical secondary structure on the MoS_2_ surface, with a non-parallel orientation. According to the ssp and ppp SFG amide I spectra, the orientation of the adsorbed hybrid peptide on MoS_2_ was determined to be 15 to 25 degrees for the alpha helix *vs.* the surface normal using the method published previously (Fig. 3).^29^ More details about the orientation determination can be found in the ESI.†

**Fig. 4:**
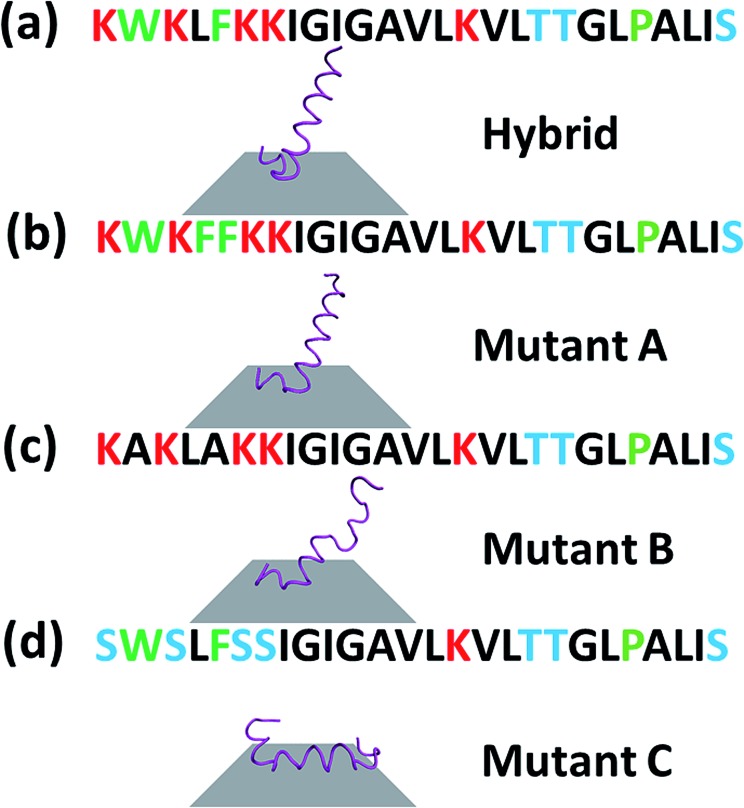
Simulation results of cecropin–melittin hybrid peptide (a), mutant A (b), mutant B (c) and mutant C (d) on an MoS_2_ surface.

To better understand the interaction that this cecropin–melittin hybrid peptide has with MoS_2_, we performed molecular dynamics simulations (see details about the simulation methods in the ESI†). Simulation results showed that the C-terminus of this peptide readily interacts with MoS_2_ and the remaining residues that are solvent accessible are at a calculated tilt angle of 20.9° from the surface normal (Fig. 4), agreeing with the experimental data quite well.

A cecropin–melittin hybrid peptide has nine amino acid residues in the C-terminus region including one aromatic group-containing residue, five non-aromatic hydrophobic groups, and three hydroxyl-containing hydrophilic residues. The N-terminus region of the cecropin–melittin hybrid peptide has two aromatic-containing amino acids, three hydrophobic (non-aromatic-containing) residues, and four charged residues. The N-terminus has one more aromatic functionality-containing residue. If the aromatic group-containing amino acid played the dominant role in surface–peptide interaction as previously reported for graphene,^13^ the peptide should interact with the MoS_2_ surface with its N-terminus. But this was not what was observed in our molecular dynamics simulations: our simulation data indicate that the C-terminus interacts with the MoS_2_ surface. We therefore believe that the aromatic amino acid/surface interaction does not play the major role in the peptide/MoS_2_ interaction. Instead, the general hydrophobic interactions play the major role: the N-terminus is more hydrophilic because of its more charged groups, and therefore prefers to stay in the aqueous environment rather than on the surface. The C-terminus overall has more hydrophobic groups, which leads to the adsorption of the C-terminus on the surface.

To further understand the peptide–MoS_2_ physico-adsorption process, we designed three cecropin–melittin hybrid peptide mutants (Fig. 2) and studied their interactions with the MoS_2_ surface. For all three mutants, only the N-terminus of the peptide was modified, which was previously identified as being primarily driven into solution for MoS_2_ interactions. Mutant A has one extra aromatic residue at the N-terminus. The goal of using this mutant was to examine whether one additional aromatic residue could increase the interaction with MoS_2_ in order to change the peptide orientation to a lying-down pose, as previously observed in peptide-graphene interactions.^13^ SFG amide I signals were successfully detected from mutant A on the MoS_2_ surface (Fig. 2b), indicating a nonparallel pose in the α-helical conformation, which matched the MD simulation data (Fig. 4b). The simulation results again indicated that the C-terminus binds to the MoS_2_ surface, while the N-terminus points away from the MoS_2_ surface into the solution. Both the SFG experimental ratio and the simulation data showed an almost identical orientation of mutant A compared to the native hybrid peptide, showing no strong interaction between the mutant A N-terminus (with one extra aromatic residue) and MoS_2_. Indeed, we found through both SFG experiments and MD simulations that even with two additional aromatic side chain-containing amino acids on the N-terminus (mutant A2), the mutant A2 could still adopt a tilting pose on MoS_2_ without lying down (Fig. S2†).

The above study on mutant A indicated that it is likely that the aromatic amino acid–MoS_2_ interaction is not stronger than the non-aromatic amino acid–MoS_2_ interaction. We wanted to know whether this is true *vice versa* and see whether the replacement of the aromatic amino acids with non-aromatic hydrophobic residues affects the peptide/MoS_2_ interaction. Therefore, the aromatic residues were all mutated to non-aromatic hydrophobic groups near the N-terminus in mutant B. SFG spectra were successfully collected from mutant B on MoS_2_ (Fig. 2c); its orientation was measured to be 15° to 25° *versus* the surface normal, similar to that deduced from the MD simulation results (α-helix with a tilt angle of 29.7°) and also similar to the case of the wild-type peptide presented above. The similar structure of mutant B on MoS_2_ to that of the wild-type hybrid indicated that the amino acids with non-aromatic functional groups do not interact with the MoS_2_ surface to a greater extent than the aromatic hydrophobic amino acids.

We then studied the effect of charged amino acids on the N-terminus by replacing charged residues with serine (mutant C). If the hydrophilic hydroxyl groups interact with water less favorably than the charged residues, mutant C might lie down on the surface. Indeed, no discernable SFG amide I signal could be detected from the interface between MoS_2_ and the mutant C solution. The absence of a SFG signal could be because (1) no peptide was adsorbed onto the MoS_2_ surface, or (2) all adsorbed peptides were lying down. To differentiate between these two possibilities, circular dichroism (CD) spectroscopy, sensitive to only secondary structure and not orientation, was used (ESI†). CD data demonstrated that mutant C was present on the MoS_2_ surface with an alpha helical secondary structure (Fig. S3†). The above finding was also validated by molecular dynamics simulation (Fig. 4d).

## Conclusion

In conclusion, we applied a unique analytical platform to combine an optical microscope with an SFG spectrometer to study peptide interactions that occur on heterogeneous MoS_2_ surfaces. This study elucidated the detailed molecular interactions between a cecropin–melittin hybrid peptide and a MoS_2_ surface. We found that the aromatic amino acids do not have a substantial effect on peptides interacting with the MoS_2_ surface. With three rationally designed peptide mutants (mutants A, B and C), more details about the peptide interactions on MoS_2_ were deduced. It was found that the charged groups in the N-terminus region are needed for the peptide to interact more favorably with the aqueous environment and ensure a “standing-up” peptide pose on MoS_2_. SFG experimental results and MD simulation results showed excellent agreement, validating the results obtained in this research: the wild-type hybrid peptide, mutant A, and mutant B were all able to interact favorably with MoS_2_*via* their C-terminus while tilting at around 20° and being solvent accessible. Mutant C, on the other hand, lay down on the MoS_2_ surface completely. This fundamental research on the hybrid peptide/MoS_2_ interactions lay a foundation for future investigations on the interactions between other peptides and MoS_2_, providing insight into the rational design of MoS_2_ based biosensors using peptides and proteins. The different mechanisms of the peptide–MoS_2_ interactions elucidated in this research, compared to the previously reported peptide–graphene interactions, clearly indicated that it is necessary to study peptide–2D material interactions when different 2D materials were chosen for sensor design. In biosensing applications, it is necessary to control the substrate surface–active biosensing unit interactions to optimize the sensing selectivity and sensitivity. This research also further demonstrated the power of using a microscope-SFG system to study heterogeneous surfaces and interfaces.

## Conflicts of interest

There are no conflicts to declare.

## Supplementary Material

Supplementary information
