# Supplementary material for: Molecular interactions between single layered MoS2 and biological molecules
Source: Chem Sci. 2017 Nov 30;9(7):1769–73. doi: 10.1039/c7sc04884j (PMC5885976; doi:10.1039/c7sc04884j)
Supplement: Supplementary file 1 [file SC-009-C7SC04884J-s001.pdf]

Electronic Supplementary Material (ESI) for Chemical Science.
This journal is © The Royal Society of Chemistry 2017

Supporting Information

Molecular Interactions between Single Layered MoS_2 and Biological Molecules

*Minyu Xiao[1], Shuai Wei[1], Yaoxin Li[1], Joshua Jasensky[1], Junjie Chen, Charles L Brooks III[1], Zhan Chen[1]**

[1]*Department of Chemistry, University of Michigan, Ann Arbor, Michigan, 48109, USA*

**Corresponding author: Zhan Chen (zhanc@umich.edu)*

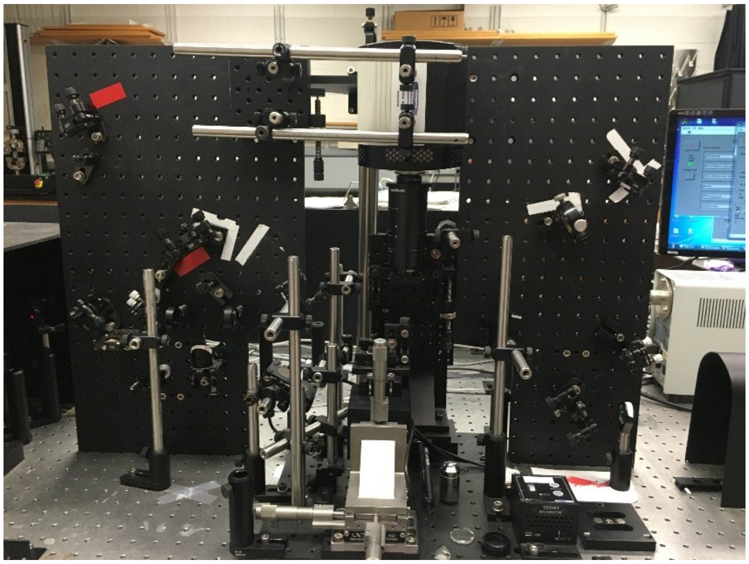

1. Microscope-SFG setup:

Figure S1 Picture of the microscope - SFG setup

2. Orientation Determination:

To quantify the orientation of a peptide on MoS_2 surface, we need to know the length of the alpha helical structure: Fig 3 displays the relationship between the measured SFG ppp vs. ssp signal strength ratio and the tilt angle of an alpha helix with different lengths. Simulation results showed the alpha helical component should contain ~19 amino acid residues, therefore we included the results for a helical structure with 17 to 21 residues. Fig 3 also shows that the variation of the amino acid number only slightly influences the relationship between the measured SFG signal strength ratio and the tilt angle of a helical structure.

From the fitted SFG ssp and ppp spectra collected from the wild type hybrid peptide on MoS_2, shown in Fig 2a, the χ_{ppp}/χ_{ssp} ratio was determined to be 1.62, corresponding to a tilt angle of 15 to 25 degrees for the alpha helix (Fig 3).

For the mutant A, the measured χ_{ppp}/χ_{ssp} ratio was found to be 1.64, similar to that measured from the wild type hybrid peptide on MoS_2. Such a ratio correlates to a tilt angle between 15° to 25° versus the surface normal.

3. SFG and simulation results of mutant A2:

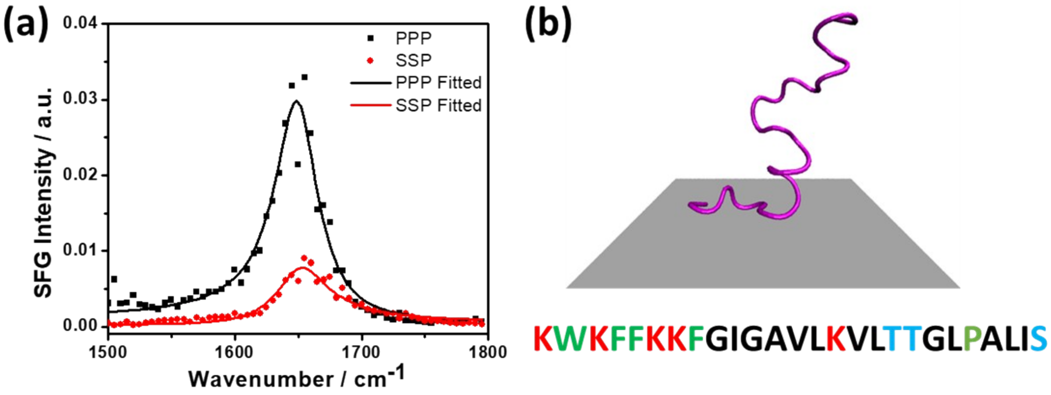

Figure S2 (a) SFG spectra collected from the interface between MoS_2 and a solution of mutant A2. (b) Simulation results of mutant A2 on MoS_2 and the sequence of mutant A2.

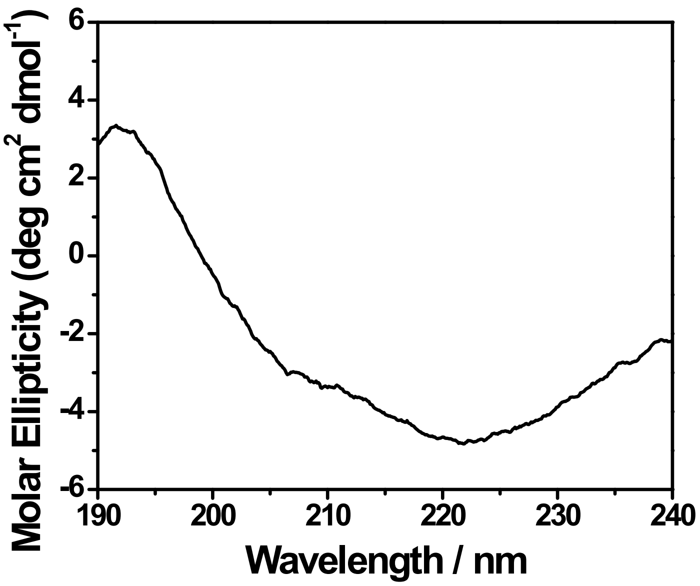

4. CD spectrum of mutant C on MoS_2:

Figure S3 CD spectrum of mutant C on MoS

5. Fitting parameters of the SFG spectra collected from the interfaces between MoS_2 and the solutions of the wild type peptide, mutant A, or mutant B

Table S1: Parameters for the SFG peak fitting

	Native	Mutant A	Mutant B
Amplitude ppp	6.4.0	3.06	1.13
Wavenumber ppp	1650	1650	1657
Width ppp	21.9	20.7	16.2
Amplitude ssp	3.9	2.14	0.71
Wavenumber ssp	1650	1650	1650
Width ssp	20.1	22.1	16.0

All final ratios showed in manuscript are corrected with Fresnel Coefficient according to previous publication.[1]

6. Simulation method

The interactions between wild-type/mutant peptides and the MoS_2 surface were simulated using a coarse-grained model developed by Wei et al.[2]. This generic surface potential model used in this research is the well-studied Karanicolas-Brooks Go-like protein model.[3, 4, 5] This protein-surface modeling system has been shown to successfully represent different material surface hydrophobicities and their quantitative effects on peptide/protein behavior at the interface. This was done through the accurate calculation of the surface binding affinities of different amino-acid residues in the peptide/protein. As shown in Equation S1, all θ's are fixed parameters obtained against a large benchmark data set[6] which were further optimized and tested in different studies. Both σ's and ε's are fixed residue based L-J parameters as

described in the model development. z's are the relative distances between residues and the material surface as measured through the simulation. The only system dependent parameter is χ_s which is assigned to be 1.5 for the MoS_2 according to the reported water contact angle of around 60 to 90 degrees.[7]

$$V_{surface} = \sum_{i}^{N} \left\{ \pi \rho \sigma_i^3 \epsilon_i \left[\theta_1 \left(\frac{\sigma_i}{z_{is}} \right)^9 - \theta_2 \left(\frac{\sigma_i}{z_{is}} \right)^7 + \theta_3 \left(\frac{\sigma_i}{z_{is}} \right)^3 - (\theta_s(\chi_s - 4.5) + \theta_p \chi_{pi}) \left(\frac{\sigma_i}{z_{is}} \right)^3 \right] \right\}, \quad (S1)$$

The parameters (shown in Table S2) used in this work were determined in the previous study.[6]

Table S2: Parameters for the surface model

θ_1	θ_2	θ_3	θ_s	θ_p
0.2340	0.4936	0.1333	0.0067	0.0333

The wild-type peptide structure was built based on an α-helical structure template. Site mutations were performed for the mutant sequences using the MMTSB tool set (*www.mmtsb.org*) with energy minimization. Since the coarse-grained model is based on the Karanicolas-Brooks Go-like model, the peptide structures were built also by the MMTSB tool set (*www.mmtsb.org*) based on the obtained atomistic structures.

The initial pose of a peptide is standing-up with C-termini close to the MoS_2 surface with a distance of 8 Å (the peptide was totally adsorbed to the surface with other initial poses). Three independent replicate simulations were performed for each system. Simulation time step was 1 fs and 10 million steps were performed for each MD simulation. All simulations were performed under NVT with the temperature at 298 K, which was maintained by 3 Nose-Hoover thermostats and the mass of $10^{-26}\ kg\text{Å}^2$.

References

1. Zou X*, et al.* Molecular Interactions between Graphene and Biological Molecules. *Journal of the American Chemical Society* **139**, 1928-1936 (2017).

2. Wei S, Knotts IV TA. A coarse grain model for protein-surface interactions. *The Journal of chemical physics* **139**, 09B631_631 (2013).

3. Karanicolas J, Brooks CL. Improved Gō-like models demonstrate the robustness of protein folding mechanisms towards non-native interactions. *Journal of molecular biology* **334**, 309-325 (2003).

4. Karanicolas J, Brooks CL. The origins of asymmetry in the folding transition states of protein L and protein G. *Protein Science* **11**, 2351-2361 (2002).

5. Karanicolas J, Brooks CL. The structural basis for biphasic kinetics in the folding of the WW domain from a formin-binding protein: lessons for protein design? *Proceedings of the National Academy of Sciences* **100**, 3954-3959 (2003).

6. Wei Y, Latour RA. Benchmark experimental data set and assessment of adsorption free energy for peptide− surface interactions. *Langmuir* **25**, 5637-5646 (2009).

7. Kozbial A, Gong X, Liu H, Li L. Understanding the intrinsic water wettability of molybdenum disulfide (MoS2). *Langmuir* **31**, 8429-8435 (2015).
